# Smoking and secondhand smoke exposure and prevalence of depressive symptoms during pregnancy in Japan: baseline data from the Kyushu Okinawa Maternal and Child Health Study

**DOI:** 10.1186/s12971-017-0139-6

**Published:** 2017-07-24

**Authors:** Yuri Kawasaki, Yoshihiro Miyake, Keiko Tanaka, Shinya Furukawa, Masashi Arakawa

**Affiliations:** 1 Department of Epidemiology and Preventive Medicine, Ehime University Graduate School of Medicine, Ehime, Japan; 2 Epidemiology and Medical Statistics Unit, Translational Research Center, Ehime University Hospital, Ehime, Japan; 3 Health Tourism Research Fields, Graduate School of Tourism Sciences, University of the Ryukyus, Okinawa, Japan

**Keywords:** Depressive symptoms, Japanese, Pregnancy, Secondhand smoke, Smoking

## Abstract

**Background:**

Epidemiological evidence on the relationship between smoking and secondhand smoke (SHS) exposure and depressive symptoms during pregnancy has been limited. The present cross-sectional study examined this issue in Japan.

**Methods:**

Between April 2007 and March 2008, 1757 pregnant women who lived in one of seven prefectures on Kyushu Island in southern Japan or in Okinawa Prefecture, an island chain in the southwest of Japan, participated in the Kyushu Okinawa Maternal and Child Health Study, a prebirth cohort study. In the present study, data on 1745 pregnant women were available for analysis. Information on smoking, SHS exposure, depressive symptoms, and potential confounding factors was obtained through a self-administered questionnaire. Depressive symptoms were defined as present when subjects had a Center for Epidemiologic Studies Depression Scale score of 16 or higher. Adjustment was made for age, gestation, region of residence, number of children, family structure, household income, education, job type, history of depression, and family history of depression.

**Results:**

The prevalence of depressive symptoms during pregnancy was 19.2%. Compared with having never smoked, both former and current smoking was independently associated with a higher prevalence of depressive symptoms during pregnancy: the adjusted odds ratios (ORs) were 1.39 (95% CI: 1.06–1.83) and 2.49 (95% CI: 1.36–4.45), respectively. Also, 3.0 to 7.9 and 8.0 or more pack-years of smoking were independently positively related to depressive symptoms during pregnancy: the adjusted ORs were 1.55 (95% CI: 1.08–2.22) and 1.97 (95% CI: 1.26–3.03), respectively (*P* for trend = 0.0005). Among the 1183 subjects who had never smoked, current SHS exposure at home was independently positively associated with depressive symptoms during pregnancy: the adjusted OR was 1.51 (95% CI: 1.003–2.30).

**Conclusions:**

Former and current smoking, 3.0 or more pack-years of smoking, and current SHS exposure at home may be positively associated with depressive symptoms during pregnancy.

## Background

A meta-analysis of longitudinal studies in adolescents suggests that the positive association between smoking and depressive symptoms is bidirectional; both the pooled estimates for smoking predicting depressive symptoms in six studies and those for depressive symptoms predicting smoking in 12 studies were statistically significant, with a stronger effect of smoking predicting depressive symptoms [1]. In 2014, Luger et al. assessed the relationship between smoking and depressive symptoms in adults using meta-analytic techniques and showed that, compared with having never smoked, former and current smoking was significantly associated with a 21% and 50% increased prevalence of depressive symptoms, respectively, using data from 78 cross-sectional studies and that smoking was significantly related to a 1.6-fold increased risk of depressive symptoms based on seven prospective studies [2]. Secondhand smoke (SHS) exposure has been found to be positively associated with depressive symptoms in several studies [3–14], while such a positive association was not observed in non-smoking adults in the Netherlands [15].

Epidemiological evidence on the relationship between smoking status and depressive symptoms during pregnancy has been limited and has produced conflicting results [9, 13, 16–20]. Three epidemiological studies showed a significant positive association between SHS exposure and depressive symptoms during pregnancy [3, 9, 13].

In light of the lack of epidemiological information regarding the relationship between smoking and SHS exposure and depressive symptoms during pregnancy in Japan, the present cross-sectional study examined this issue using baseline data from the Kyushu Okinawa Maternal and Child Health Study (KOMCHS).

## Methods

### Study population

The KOMCHS is an ongoing prospective prebirth cohort study. Details of the baseline survey of the KOMCHS have been described elsewhere [21]. Eligible study subjects were pregnant women who lived in one of seven prefectures on Kyushu Island in southern Japan or in Okinawa Prefecture, an island chain in the southwest of Japan. Between April 2007 and March 2008, we requested that 131 obstetric hospitals in Fukuoka Prefecture, the largest prefecture on Kyushu Island (with a total population of approximately 5.04 million), provide as many pregnant women as possible with a set of leaflets explaining the KOMCHS, an application form to participate in the study, and a self-addressed and stamped return envelope. Between May 2007 and March 2008, we also requested that 40 obstetric hospitals in Okinawa Prefecture, with a total population of nearly 1.37 million, provide as many pregnant women as possible with the same documents. Between August 2007 and March 2008, pregnant women living in six prefectures on Kyushu Island other than Fukuoka Prefecture, with a total population of approximately 8.22 million, were also provided with the same documents at 252 obstetric hospitals. Pregnant women who were willing to participate in the study returned the application form containing a written description of their personal information to the data management center. Upon receiving this personal information, research technicians provided each woman with a detailed explanation of the KOMCHS via telephone and sent them a self-administered questionnaire after obtaining their agreement. A total of 1757 pregnant women between the 5th and 39th weeks of pregnancy gave their written informed consent to participate in the KOMCHS and completed the baseline survey. After 12 pregnant women were excluded because of incomplete data on the variables under study, data on 1745 pregnant women were available for analysis. The KOMCHS was approved by the ethics committees of the Faculty of Medicine, Fukuoka University and Ehime University Graduate School of Medicine.

### Measurements

In the baseline survey, each participant filled out a two-part questionnaire and mailed it to the data management center. Research technicians completed missing or illogical data by telephone.

The first part of the questionnaire elicited information on age, gestation, region of residence, number of children, family structure, household income, educational level, employment status, personal history of doctor-diagnosed depression, family history of depression, smoking habits, and secondhand smoke exposure at home and at work. Cumulative exposure to cigarette smoking was summarized by multiplying the average number of packs smoked per day (cigarettes smoked per day divided by 20) by the number of years smoked (pack-years of smoking), regardless of whether smoking status was former or current. SHS exposure at home and at work was assessed, respectively, by the following questions: “Have you ever been exposed to smoke from family members at home?” and “Have you ever been exposed to smoke at your workplace?” Employment status was elicited for the year in which the questionnaire was conducted and for the previous year; women were classified as unemployed if they were unemployed both in the year in which the questionnaire was completed and in the preceding year. A family history of depression was considered to be present if one or more parents or siblings of the study subjects had been diagnosed with depression by a physician.

Depressive symptoms were assessed using a Japanese version [22] of the Center for Epidemiologic Studies Depression Scale (CES-D) [23], which was included in the first part of the questionnaire. This scale consists of 20 questions addressing six symptoms of depression, including depressed mood, feelings of guilt or worthlessness, helplessness or hopelessness, psychomotor retardation, loss of appetite, and sleep disturbance experienced during the preceding week. Each question is scored on a scale of 0 to 3 according to the frequency of the symptoms, and the total CES-D score ranges from 0 to 60. The criterion validity of the CES-D scale has been well established in adult Western [23] and Japanese [22] populations. Consistent with the validation studies, we defined depressive symptoms as present when a subject had a CES-D score ≥ 16.

The second part of the questionnaire was a diet history questionnaire. Data regarding diet were not used in the present study.

### Statistical analysis

Age, gestation, region of residence, number of children, family structure, household income, education, job type, history of depression, and family history of depression were selected a priori as potential confounding factors. Age and gestation were used as continuous variables.

Crude odds ratios (ORs) and their 95% confidence intervals (CIs) for associations between smoking and SHS exposure and the prevalence of depressive symptoms during pregnancy were estimated with logistic regression analysis. To adjust for potential confounding factors, multiple logistic regression analysis was used. Trend of association was assessed using a logistic regression model assigning consecutive integers to the categories of the exposure variables. The analyses regarding the relationship of SHS exposure at home and at work with depressive symptoms during pregnancy were limited to the 1183 subjects who had never smoked. All analyses were conducted using the SAS software package version 9.4 (SAS Institute, Inc., Cary, NC, USA).

## Results

The prevalence of depressive symptoms during pregnancy was 19.2% among the 1745 pregnant women. The mean age and gestation at baseline of the study subjects were 31.2 years and 18.5 weeks, respectively (Table 1). Approximately 60% had one or more children, roughly 5% had a personal history of depression, and 10% had a family history of depression.

**Table 1:** Distribution of selected characteristics in 1745 women, Kyushu Okinawa Maternal and Child Health Study, Japan, April 2007 to March 2008

Variable	*n* (%)
Age, years, mean ± SD	31.2 ± 4.4
Gestation, weeks, mean ± SD	18.5 ± 5.4
Region of residence	
Fukuoka Prefecture	970 (55.6)
Other than Fukuoka Prefecture in Kyushu	593 (34.0)
Okinawa Prefecture	182 (10.4)
Number of children	
0	703 (40.3)
1	690 (39.5)
≥ 2	352 (20.2)
Nuclear family structure	1474 (84.5)
Family income, yen/year	
< 4,000,000	633 (36.3)
4,000,000–5,999,999	620 (35.5)
≥ 6,000,000	492 (28.2)
Education, years﻿	
< 13	430 (24.6)
13–14	575 (33.0)
≥ 15	740 (42.4)
Job type^a^	
Unemployed	707 (40.5)
Professional or technical	433 (24.8)
Clerical or related occupation	328 (18.8)
Sales	83 (4.8)
Service	115 (6.6)
Production	51 (2.9)
Others^b^	28 (1.6)
History of depression	84 (4.8)
Family history of depression	175 (10.0)

^a^ Full-time or part-time employment in the year when the first questionnaire was conducted or in the previous year

^b^Management; protection services; farming, fishing, or forestry; transportation or communications; or construction

Table 2 shows crude and adjusted ORs and 95% CIs for depressive symptoms during pregnancy in relation to smoking status. Compared with having never smoked, both former and current smoking was significantly associated with the presence of depressive symptoms during pregnancy. After adjustment for the confounding factors under study, the positive associations remained significant, albeit slightly attenuated: the adjusted ORs were 1.39 (95% CI: 1.06–1.83) and 2.49 (95% CI: 1.36–4.45), respectively. Compared with having never smoked, 3.0 to 7.9 and 8.0 or more pack-years of smoking were independently positively related to the prevalence of depressive symptoms during pregnancy, showing a clear exposure-response relationship with the cumulative consumption of cigarettes in the multivariate model: the adjusted ORs were 1.55 (95% CI: 1.08–2.22) and 1.97 (95% CI: 1.26–3.03), respectively (*P* for trend = 0.0005).

**Table 2:** Odds ratios (ORs) and 95% confidence intervals (CIs) for depressive symptoms during pregnancy in relation to smoking status in 1745 pregnant women, Kyushu Okinawa Maternal and Child Health Study, Japan, April 2007 to March 2008

	Prevalence (%)	Crude OR (95% CI)	Adjusted OR (95% CI)^a^
Smoking status			
Never	196/1183 (16.6)	1.00	1.00
Former	118/503 (23.5)	1.54 (1.19–1.99)	1.39 (1.06–1.83)
Current	21/59 (35.6)	2.78 (1.57–4.80)	2.49 (1.36–4.45)
Pack-years of smoking			
None	196/1183 (16.6)	1.00	1.00
0.05–2.9	45/207 (21.7)	1.40 (0.96–2.00)	1.18 (0.80–1.72)
3.0–7.9	56/222 (25.2)	1.70 (1.20–2.37)	1.55 (1.08–2.22)
≥ 8.0	38/133 (28.6)	2.01 (1.33–3.00)	1.97 (1.26–3.03)
*P* for trend		< .0001	0.0005

^a^Adjusted for age, gestation, region of residence, number of children, family structure, family income, education, job type, history of depression, and family history of depression

Table 3 shows the association between SHS exposure and depressive symptoms during pregnancy among the 1183 subjects who had never smoked. Current, but not former, SHS exposure at home was independently associated with a higher prevalence of depressive symptoms during pregnancy: the adjusted OR was 1.51 (95% CI: 1.003–2.30). A relationship between current SHS exposure at work and depressive symptoms during pregnancy fell just short of the significance level after adjustment for confounders.

**Table 3:** Odds ratios (ORs) and 95% confidence intervals (CIs) for depressive symptoms during pregnancy in relation to secondhand smoke exposure in 1183 pregnant women who had never smoked, Kyushu Okinawa Maternal and Child Health Study, Japan, April 2007 to March 2008

	Prevalence (%)	Crude OR (95% CI)	Adjusted OR (95% CI)^a^
Secondhand smoke exposure at home			
Never	48/351 (13.7)	1.00	1.00
Former	72/436 (16.5)	1.25 (0.84–1.86)	1.18 (0.79–1.78)
Current	76/396 (19.2)	1.50 (1.01–2.23)	1.51 (1.003–2.30)
Secondhand smoke exposure at work			
Never	73/503 (14.5)	1.00	1.00
Former	99/550 (18.0)	1.29 (0.93–1.80)	1.05 (0.74–1.50)
Current	24/130 (18.5)	1.33 (0.79–2.19)	1.75 (0.995–3.01)

^a^ Adjusted for age, gestation, region of residence, number of children, family structure, family income, education, job type, history of depression, and family history of depression

## Discussion

Our results regarding active smoking are in partial agreement with those of a meta-analysis using data from 78 cross-sectional studies in adults showing significant positive associations between former and current smoking and depressive symptoms. A cross-sectional study of 236 US pregnant women found significant positive relationships between active smoking and each of 10 items of the Edinburgh Postnatal Depression Scale (EPDS) [9]. In a cross-sectional study of 462 minority pregnant women in the USA who had smoked, significant positive associations were reported between current smoking and mild and moderate-to-severe depressive symptoms as assessed via the Beck Depression Inventory Fast Screen [13]. A significant positive association between smoking during pregnancy and depressive symptoms during pregnancy based on the EPDS was shown in a cross-sectional study of 1264 Brazilian women [19]. A cross-sectional study of 487 pregnant Norwegian women observed significant positive relationships between current and former smoking and depressive symptoms based on the CES-D [20]. These findings are in partial agreement with our results. No significant association was observed between smoking status and depressive symptoms based on the EPDS in a cross-sectional study of 921 pregnant Puerto Rican and Dominican women in the USA [16] and in a cross-sectional study of 2203 Medicaid-eligible pregnant US women [17]. A cross-sectional study of 916 pregnant Romanian women found no relationship between smoking during pregnancy and depressive symptoms based on the Patient Health Questionnare-2 [18]. These findings are at variance with our results. In a prospective study of 533 Taiwanese couples, paternal smoking in the mother’s presence significantly increased maternal depressive symptoms during pregnancy based on the EPDS [3]. Significant positive relationships were shown between SHS exposure and each of 10 items of the EPDS in a previously cited cross-sectional study of 236 pregnant US women [9]. In a cross-sectional study of 467 minority pregnant women in the USA who had never smoked, a significant positive association was found between SHS exposure and moderate-to-severe, but not mild, depressive symptoms based on the Beck Depression Inventory Fast Screen [13]. These findings are in partial agreement with our results.

The biological mechanism that may link smoking and SHS exposure to depressive symptoms is unclear. An animal study in rats demonstrated that exposure to nicotine during adolescence, but not during adulthood, leads to both a depression-like state manifested in decreased sensitivity to natural reward and an enhanced sensitivity to stress- and anxiety-eliciting situations later in life [24]. Dopaminergic-system dysregulation may be linked to depressive symptoms [25]. Another animal study in rats found that nicotine and tobacco particulate matter affect the function and expression of the dopamine and norepinephrine transporters [26]. Li et al. reported that chronic nicotine and smoking exposure increases dopamine transporter mRNA expression in the rat midbrain [27]. Nicotinic acetylcholine receptors are located in areas of the brain implicated in depression, and are involved in regulating neurobiological systems implicated in depression, including monoamine neurotransmitters, the hypothalamic-pituitary-adrenal axis, and certain inflammatory processes [28]. With respect to the observed positive relationship between SHS exposure at home and depressive symptoms during pregnancy, some unknown factors associated with SHS exposure at home may have confounded the observed relationship. For example, SHS exposure at home is likely to be linked to stress or poor socioeconomic status, which may be associated with depressive symptoms during pregnancy.

There are several methodological limitations to our study. To begin with, the cross-sectional nature of the present study prevents us from drawing conclusions about causality. Depressive women might be more at risk of smoking and SHS exposure during pregnancy.

Smoking and SHS exposure were assessed based on the participants’ questionnaire responses; data on objective measurements such as salivary, serum, or hair cotinine levels were not available. Some pregnant women might have deliberately concealed their smoking habits in order to give a socially desirable response. Data on the number of cigarettes involved in SHS exposure were not available. The consequence of non-differential exposure misclassification would bias the estimates of the association between exposure and outcome towards the null.

Depressive symptoms were assessed using the CES-D scale rather than structured diagnostic interviews. The CES-D includes questions on physical symptoms such as fatigue and physical discomfort, which are also typical complaints of pregnancy; the consequence of this symptom overlap could have been an overestimation of depression. The prevalence of depressive symptoms in the present study was, however, lower than that in a representative sample of the Japanese general population: the prevalence of depressive symptoms (CES-D score of ≥16) was 30.7% in 2315 women aged 30–39 years [29]. Moreover, our study subjects took part in the baseline survey at various points between the 5th and 39th week of pregnancy: 594 (34.0%), 1005 (57.6%), and 146 (8.4%) participants completed the baseline survey in the first (≤ 15 weeks’ gestation), second (16–27 weeks’ gestation), and third (≥ 28 weeks’ gestation) trimesters, respectively. Therefore, it is difficult to accurately estimate the incidence and prevalence of depressive symptoms during pregnancy. The possibility of non-differential outcome misclassification would have been an underestimation of values in our results. The results of a sensitivity analysis restricted to 1005 pregnant women who completed the baseline survey in the second trimester were similar to those in the overall analysis: the adjusted OR was 2.37 (95% CI: 1.002–5.25) for current smoking, 1.98 (95% CI: 1.03–3.68, *P* for trend = 0.03) for 8.0 or more pack-years of smoking, 1.44 (95% CI: 0.81–2.61) for SHS exposure at home, and 2.23 (95% CI: 1.08–4.52) for SHS exposure at work; data on 683 women who had never smoked were used in the analyses regarding SHS exposure.

We could not calculate the participation rate because we do not have exact figures for the number of pregnant women who were provided with a set of leaflets explaining the KOMCHS, an application form, and a self-addressed and stamped return envelope by the 423 collaborating obstetric hospitals. We were not able to assess the differences between participants and non-participants because information on personal characteristics such as age, socioeconomic status, and history of depression was not available for non-participants. Our subjects were probably not representative of Japanese women in the general population, however. For example, a population census conducted in 2000 in Fukuoka Prefecture found that the percentages of women aged 30 to 34 years with <13, 13–14, ≥ 15, and an unknown number of years of education were 52.0%, 31.5%, 11.8%, and 4.8%, respectively [30]. The corresponding figures for this study were 24.6%, 33.0%, 42.4%, and 0.0%, respectively. Thus, our study subjects were more educated and probably more aware of health topics than women in the general population.

Although adjustment was made for several confounding factors, residual confounding effects could not be ruled out.

## Conclusions

The present cross-sectional study in Japan showed that former and current smoking, 3.0 or more pack-years of smoking, and SHS exposure at home were independently associated with a higher prevalence of depressive symptoms during pregnancy. Further evidence from prospective cohort studies with a more precise assessment of smoking, SHS exposure, and depressive symptoms is required to draw a conclusion as to whether smoking and SHS exposure are risk factors for depressive symptoms during pregnancy.

## Abbreviations

CES-D: Center for Epidemiologic Studies Depression Scale
CI: Confidence interval
EPDS: Edinburgh Postnatal Depression Scale
KOMCHS: Kyushu Okinawa Maternal and Child Health Study
OR: Odds ratio
SHS: Secondhand smoke
